# Parental perspectives of prenatal counseling and decision making for termination or hospice birth due to fetal congenital heart defects

**DOI:** 10.1371/journal.pone.0353868

**Published:** 2026-07-30

**Authors:** Grace Knowles, Diana Madden, Leonardo Barrera, Harshman Sihra, Lynn M. Yee, Jessica T. Fry, William A. Grobman, Natalia Henner, Angira Patel, Sheetal Patel, Joyce L. Woo

**Affiliations:** 1 Division of Palliative Care, Ann and Robert H. Lurie Children’s Hospital of Chicago, Chicago, Illinois, United States of America; 2 Division of Cardiology, Ann and Robert H. Lurie Children’s Hospital of Chicago, Chicago, Illinois, United States of America; 3 Smith Child Health Outcomes Research and Evaluation Center, Stanley Manne Children’s Research Institute at Lurie Children’s, Chicago, Illinois, United States of America; 4 University of Cincinnati, Cincinnati, Ohio, United States of America; 5 Department of Obstetrics and Gynecology, Northwestern University Feinberg School of Medicine, Chicago, Illinois, United States of America; 6 Division of Neonatology, Ann and Robert H. Lurie Children’s Hospital of Chicago, Chicago, Illinois, United States of America; 7 Department of Pediatrics, Northwestern University Feinberg School of Medicine, Chicago, Illinois, United States of America; 8 Department of Obstetrics and Gynecology, The Warren Alpert Medical School of Brown University, Providence, Rhode Island, United States of America; University of Ghana, GHANA

## Abstract

**Objective:**

To evaluate perspectives of counseling and decision making among birthing parents who chose termination or hospice birth due to a fetal congenital heart defect (CHD).

**Methods:**

Qualitative analysis of semi-structured interviews with birthing parents who chose either termination or hospice birth due to severe fetal CHD. Participants were recruited from a purposive sample of those who received multidisciplinary counseling at a tertiary congenital heart surgical center between 2019–2023. Thematic analysis was conducted in five stages, using an integrated deductive-inductive approach.

**Results:**

In total, 20 individuals were eligible; 10 completed interviews. Identified themes were categorized in two domains: Experiences During Counseling and Individual Factors Affecting Decision Making. Themes in the first domain included: areas for improvement during counseling, perceived strengths of counseling, patient attributes affecting counseling, and views on provider recommendations. Themes in the second domain included: emotions affecting decision making, value for reproductive autonomy, family factors, internal factors, outside input, and desire for resources.

**Conclusions:**

In this study, birthing parents who chose termination or hospice for fetal CHD appreciated information extending beyond clinical outcomes to include family and financial impacts. Their value for reproductive autonomy reflects the need for clinicians to gain skills in empathetic and neutral counseling of all management options.

## Background

Congenital heart defects (CHDs) are the most common birth defects in the United States, impacting about 1/100 live births [1]. Of these, ~ 25% are severe CHDs, requiring neonatal surgical intervention for survival [2]. Prenatal diagnosis allows families to choose among management options, depending on the local availability of these options [3]: termination of pregnancy (hereafter, “termination”), plan for hospice birth (i.e., perinatal hospice, palliative birth plan, or comfort care without surgical intervention), or plan for postnatal surgical intervention [4,5]).

Understanding how to best counsel parents following prenatal diagnosis of severe CHD is a significant area of fetal cardiology research [6–8]. Although several studies have examined patient experiences with prenatal counseling [9–11], most analyses have studied those who chose postnatal surgical intervention [12–18]. This bias results in inadequate information regarding perspectives and decision-making of termination and hospice birth counseling. In the extreme, this bias may lead to perceptions that termination discussions are insensitive, or incomplete information about all management options [13,17,19,20]. Thus, understanding perspectives of individuals who have experienced termination or hospice birth is a key knowledge gap in developing counseling practices that present all management options, a key component of fully informed consent.

We aimed to evaluate perspectives on counseling and decision making for termination or hospice birth due to severe fetal CHD, among birthing parents who received multidisciplinary counseling at a tertiary center in the United States where both termination and hospice birth services are legal and available.

## Methods

### Sampling and recruitment

This is a prospective qualitative analysis that examined the experiences of birthing parents who had a fetus with severe CHD and either terminated their pregnancy or elected for a hospice birth plan at Ann & Robert H. Lurie Children’s Hospital of Chicago (hereafter, “Lurie Children’s”) or Northwestern University Prentice Women’s Hospital. Severe CHD is defined as defects that require neonatal intervention for survival (e.g., single ventricle). Eligibility was determined through purposive sampling of birthing parents who had received multidisciplinary counseling for severe fetal CHD between 6/1/2019 and 3/31/2023 at Lurie Children’s. We did not recruit patients who received multidisciplinary counseling before 2019 to avoid recall bias. We chose March 2023 as the upper bound to allow at least nine months of bereavement time, between receipt of multidisciplinary counseling and study recruitment [21,22].

This purposive sample includes individuals who received counseling before and after the 2022 *Dobbs v. Jackson Women’s Health Organization* (*Dobbs*) ruling, which revoked the constitutional right to termination and returned authority to regulate termination to individual states, thus impacting access to care in certain geographic areas of the United States [23,24]. In Illinois, the Illinois Reproductive Health Act, signed in 2019, was strengthened after *Dobbs*, making Illinois a destination for out-of-state patients seeking termination [25].

Multidisciplinary counseling at Lurie Children’s entails a combined meeting with specialists from fetal cardiology, genetics, maternal-fetal medicine, neonatology, perinatal palliative care, pediatric cardiac intensive care, pediatric cardiac surgery, and social work, typically within four weeks of receiving a diagnosis of severe fetal CHD. Not all individuals who receive multidisciplinary counseling are offered a hospice birth plan; single ventricle physiology would be offered, while biventricular physiology (e.g., transposition of the great arteries) would not. Individuals who elected for postnatal surgical intervention, or who did not receive multidisciplinary counseling at this academic center, were ineligible for recruitment. All individuals who received multidisciplinary counseling had previously received initial counseling from a fetal cardiologist, social worker, and fetal nurse coordinator prior to their multidisciplinary counseling session, in accordance with standard practice [7]. Counseling providers varied between multidisciplinary counseling sessions, except for social work and palliative care, for which the same individuals were present for each session.

Eligible participants were contacted by email and telephone at least three times about the study and provided written or verbal consent prior to participation. All participants completed a questionnaire pertaining to demographic data prior to their interview; no electronic health record data were used in analysis. All participants were offered a $100 gift card for their participation. This compensation value was determined based on standard practice for qualitative interview research on sensitive topics at our institution.

### Interviews

The interview guide (**Supplemental Document 1**) contained 22 open-ended questions that addressed the prenatal counseling experience, decision making about management, and for those who underwent a hospice birth plan, the postnatal hospice experience. The interview guide was created with collaborative input from fetal cardiologists (JW, SP, AP), neonatologists (JTF, NH), palliative care specialists (GK, JTF, NH), maternal-fetal medicine specialists (WG, LY), a bioethicist (AP), and two parents from the purposive sample.

An *a priori* target recruitment of 10 interviews was established by the author team, unless saturation was not achieved with this goal. Saturation, defined by the themes identified by the interviewer following their own review of interview transcripts prior to coding, was achieved after 7 interviews. However, given that primary and secondary coding of the transcripts would be conducted by three different analysts (and not the interviewer), an additional three interviews were conducted in case that additional themes might be identified. The recruitment period was September 1, 2023 to March 30, 2024. Patient interviews were conducted between October 2023 and April 2024.

Recruited participants underwent individual virtual interviews with a trained research team member (GK). The interviewer is an advanced nurse practitioner specializing in perinatal palliative care who was present at each participant’s multidisciplinary meeting and thus had existing rapport and experience with discussing highly sensitive topics with an empathic, nonjudgemental, and open-ended style. Each interview lasted 60–90 minutes. The lack of blinding between interviewer and participants was to foster openness given the complexity and sensitive nature of the subject matter, though social desirability in patient responses was possible. The interviewer intentionally adhered closely to the interview guide to mitigate bias and ensure consistency/dependability across interviews. The interviewer identifies as a cisgender, non-minority woman without lived experience of the topic. Although involved in patient care, she did not have authority over neonatal disposition after birth. All interviews were recorded, professionally transcribed, and deidentified prior to analysis.

### Analysis and ethical oversight

Thematic analysis was conducted in five stages, using a combined deductive-inductive approach. First, a deductive codebook was created by three team members (DM, LB, JW) who identified initial codes based on the interview guide. Next, comparison coding was conducted based on three randomly chosen interviews, and the codebook was iteratively refined based on discussion of comparison coding (DM, LB, JW). Third, primary and secondary coding of each of the 10 interview transcripts were performed, with additional refinement of the codebook, and an initial narrative report outlining codes was written (DM, LB). Fourth, four team members independently reviewed the narrative report, and arranged the content into themes (GK, HS, JW, LY). Finally, to enhance credibility and confirmability, remaining team members (JTF, WAG, NH, AP, SP) addressed reflexivity with refinement and re-aggregation of themes.

Analysis was conducted using Dedoose (Version 9.0.46, 2021. Los Angeles, CA: SocioCultural Research Consultants, LLC). To protect participant privacy, limited demographic and clinical descriptive data are presented. To further ensure confirmability and authenticity, each subtheme was described with a direct interview quotation from three separate participants – one within the text, and two within the tables. The analysis is presented in accordance with the SRQR reporting guidelines **(Supplemental Document 2)**. The study was approved by the Institutional Review Board at Lurie Children’s (2023–6213).

## Results

In total, 20 individuals were eligible for study recruitment. Of the 10 individuals who participated in interviews, seven elected for termination and three elected for hospice birth. The average time between multidisciplinary counseling and participation in the study was 2.9 (standard deviation 1.1) years. Six had fetuses with single ventricle CHD and were thus offered hospice birth plans. Nine identified as non-Hispanic White and all had at least a bachelor’s degree. All individuals were married and employed; nine had commercial insurance and eight had another living child. Six individuals identified as atheist, agnostic, or non-religious, while four individuals identified as Christian or Catholic. Among the three individuals who elected for hospice birth, one pregnancy ended in stillbirth, one experienced a live birth where the newborn died in the hospital, and one experienced home hospice care.

Themes were categorized into two domains: experiences during counseling (Table 1**),** and individual considerations in decision making (Table 2). Initial analyses identified that participants described counseling experiences that were barriers (i.e., factors that hindered decision making), facilitators (i.e., factors that enabled decision making), or both. In contrast, individual considerations in decision making were not as clearly delineated as barriers or facilitators, therefore, themes in this domain are described without use of this framework.

**Table 1:** Barriers and facilitators of decision making related to prenatal counseling.

Theme	Subtheme	Excerpt 1	Excerpt 2
**Barriers**			
Areas for improvement in counseling practices	Insufficient discussion about termination or hospice birth	*“I didn’t really feel like [outside hospital] talked a lot about palliative care.”* (Hospice)	*“[gynecologist] was the only one that told me if you don’t need to continue with this pregnancy if you don’t….they [multidisciplinary team] didn’t really talk much to me about that [hospice]”* (Termination)
	Perceived lack of urgency	*“I was waiting for all this information [amniocentesis results] and then I didn’t even get it until after [termination]....”* (Termination)	*“... it was three weeks of a nightmare to be honest because we had to wait for a long time to talk to different people.”* (Termination)
	Inconsiderate behaviors	*“[doctor] came into the meeting later and clapped my husband on the shoulder and shook his hand and said congratulations…that really sucked…”* (Termination)	*“[doctor said] that’s an easy fix... I don’t want to hear that because then it’s, it opens up, this [diagnosis of fetal CHD] isn’t as bad as I think it is.”* (Termination)
	Lack of acknowledgment of impact of fetal CHD on family unit	*“I had a three-year-old at the time…but I don’t really feel like the care team talked about that.”* (Termination)	*“I don’t think there was any mention of our older child in terms of how this would impact her life.”* (Termination)
	Insufficiently specific or transparent language	*“I think you have to be clear about what it [termination or interruption] is.”* (Termination)	*“I think they [sonographer and cardiologist] can be a little bit more human and say... we think there might be something.”*(Termination)
**Facilitators**			
Perceived strengths of counseling practices	Value of multidisciplinary counseling	*“Having that point person [nurse coordinator] was actually very helpful... just having someone who was helping with all of that [logistics of scheduling] was good.”* (Termination)	*“... the genetic counselors are also really helpful to talk to… the general, multidisciplinary team… I don’t think we could ask for anything better to be honest.”* (Termination)
	Transparency about outcomes	*“I guess the certainty of knowing that there was a just terribly difficult path no matter what, that’s the only certainty we really had.”* (Termination)	*“I remember the cardiologist saying…I wouldn’t be offering this [hospice birth] to you if the condition wasn’t as serious as it is, and...something I thought about as we made our decision.”* (Hospice)
	Respect for patient autonomy	*“I do think each person [on multidisciplinary team] said, hey, this is my specialty, would you be interested in hearing?”* (Termination)	*“No one dwelled on it [termination] in any particular way and I’m grateful that they didn’t, because at that point I didn’t want to consider that as an option.”* (Termination)
	Patience and understanding	*“They [multidisciplinary counseling team] let us take all the time we wanted, ask any question, there are no dumb questions.”* (Termination)	*“...my husband said, some kind of insensitive thing in processing all of this, everyone was very patient... I didn’t feel like they were judging him...”* (Hospice)
	Nonverbal communication practices	*“…it does support families to give a little bit of privacy and a little bit of insulation [on day of counseling] if possible.”* (Termination)	*“I could tell something was wrong but no one was telling me what was happening. I mean, I could see the faces of the people….”* (Termination)
	Use of written materials	*“He [cardiologist] drew pictures, he showed us videos, that was all really helpful.”* (Termination)	*“I had the packet and it had everybody’s names...and everybody was really helpful with who I had to get in touch with....”* (Termination)
**Context-Dependent Barrier or Facilitator**			
Patient attributes specifically affecting counseling	Emotions: shock and information overload (barrier)	*“I feel like I kind of blacked out a lot of the meeting after the diagnosis.”* (Termination)	*“…it was just so much. I mean you could lay out thousands of different paths.”* (Termination)
	Previous knowledge, or attitudes toward hospice birth (barrier and facilitator)	*“I cannot do that [hospice]. I couldn’t be pregnant for another several more months …and then have to say goodbye to him.”* (Termination)	*“I had a pretty full picture of what it [hospice] would be. Maybe just not the specifics.”* (Hospice)
	Previous knowledge, or attitudes toward termination (barrier)	*“ …we never would have even thought that we would terminate... I remember not even wanting to consider it.”* (Termination)	*“No, it [termination] never was [a consideration]…II grew up being told that abortion was bad and everything else.”* (Hospice)
Views on provider recommendations	Undesired recommendation from provider (barrier)	*“[termination] wasn’t really presented to me as an option, which also just made me feel like, is this something that other people just suck it up or it’s not really that bad?’”* (Termination)	*“she [partner] and I left feeling pressured to terminate the pregnancy. That was her [obsetrician’s] recommendation…I felt like I wouldn’t have been supported in my labor and delivery plan...”* (Hospice)
	Desire for provider recommendation (facilitator)	*“Sometimes you just want an answer and you’re like, what do I do? I’m so lost.”* (Termination)	*“I would have been totally fine with [provider recommendation]… it would have given us maybe a little bit less guilt if we knew that the doctors also strongly felt a certain way.”* (Termination)

**Table 2:** Individual factors affecting decision making for termination or hospice birth.

Theme	Subtheme	Excerpt 1	Excerpt 2
Emotions affecting decision making following counseling	Guilt	*“...it’s like each option is selfish and each option is selfless…that part was really hard for me.”* (Hospice)	*“Because I mean, I did have a lot of guilt. I’m Catholic.”* (Hospice)
	Uncertainty	*“The uncertainty was definitely a really difficult piece of it..”* (Termination)	*“But yeah, you never know. Maybe he would have beat the odds.”* (Termination)
	Fear of stigma	*“I remember being very concerned about like people not knowing details of what was going on at work, at least... because I just didn’t want to feel judged...”* (Termination)	*“She [counseling provider] suggested me to say that I lost the baby and not that it was something that…she just told me just to avoid problems with people...”* (Termination)
Value for reproductive autonomy	Reproductive justice	*“I am thankful that I did have a choice [to terminate]…I also just felt an incredible amount of privilege or anger that other people don’t have that…”* (Termination)	*“…right when Roe v. Wade was struck down, I was in the hospital reading about women who were in my exact same situation would not have the choice that I was able to make.”* (Termination)
	Shifts in opinions about termination	*“Initially, it [termination] was something I did not want to consider at all..”* (Termination)	*“I have changed my, I don’t know, my thought process on it [termination] and I definitely fully support any person that chooses it or even considers it...”* (Hospice)
Family factors	Spousal disagreement	*“I was on board with doing palliative care, but then my husband wasn’t...”* (Hospice)	*“My husband did not agree with the decision. So, we had a lot of conflict in between us about it.”* (Termination)
	Baby’s suffering	*“We just wanted to do whatever would kind of cause him the least amount of pain and suffering.”* (Termination)	*“So then are we going to make [baby with CHD]] suffer unnecessarily, right?”* (Hospice)
	Baby’s long-term outcomes	*“...I was looking at those blogs of the kids that are actually doing relatively well… so in the best case scenario, this is still a lot to put on a kid.”* (Termination)	*“..what is their physical health, you want to know their mental health and their life as an adult. So, there’s different stages that you’re worried about them.”* (Termination)
	Effects on living children	*“That [impact on family life] was an extremely important part of our decision, this was his [living child’s] life too...”* (Termination)	*“...something that we thought about also our older daughter’s life.”* (Termination)
	Financial consequences	*“I just didn’t know what our finances looks like going forward.”* (Termination)	*“Financial was also tremendous... and of course, it would be extremely expensive should we have to repeated open heart surgeries...”* (Termination)
	Insurance and access to care	*“We were on Medicaid at that time…but that wasn’t gonna last forever…”* (Termination)	*“So insurance was the only, I think thing that was making that process [decision making about the pregnancy] difficult…”* (Termination)
Internal factors	Personal risk tolerance	*“We are risk averse people in general... just knowing that there were those super negative options, that was enough.”* (Termination)	*“I think ultimately that’s kind of the inability to really have a clear sense of the future.”* (Termination)
	Religious beliefs	*“And accepting God’s will for his life, the life that God gave him meaning…it [the decision for hospice birth] felt right, ultimately.”* (Hospice)	*“Because I mean, I did have a lot of guilt. I’m Catholic…”* (Hospice)
	Shifts in personal priorities	*“That loss of identity, I think, is really hard… I knew just flip flopping my whole identity was going to be tough too.”* (Termination)	*“There was also the just lifestyle concerns we live. We travel quite a bit or we have family abroad and we just felt like it would just be a very different.”* (Termination)
Outside input	Family	*“He [patient’s uncle] was saying that [spouse] would have to quit her job and they’d be at the hospital constantly.”* (Termination)	*“...[family member] was a secretary in a pediatric intensive care unit her whole life... and I could see that she was more leaning towards not having the child.”* (Termination)
	Friends or community	*“…but there was a lot of questions and I would say judgment from, not family, but friends. Like, why are you doing that? Why aren’t you terminating?”* (Termination)	*“And she [neighbor who is cardiologist] told me not so much because they’re sick a lot. And to me that was a deciding factor.”* (Termination)
	Blogs and social media	*“but for me, looking at some of those blogs and some of those things… I just knew that it [surgical palliation] wasn’t what I wanted for my child.”* (Termination)	*“I joined the other Facebook groups that have kids that have hypoplastic left heart that they chose the surgery route...that was ultimately why we decided to do palliative care.”* (Hospice)
Desire for resources	Experiences with termination or hospice birth	*“I think that that would be super helpful just to have to talk to other people who have been through something similar.”* (Termination)	*“Actually going through [blog posts] and describing kind of the termination and after termination; that was really helpful… that wasn’t necessarily from the medical team.”* (Termination)
	Experiences of children living with CHD	*“They [multidisciplinary team] put me in touch with another mom whose son had hypoplastic left heart.”* (Termination)	*“I found there was a group [of] families that have kids with congenital heart disease that you can join. I didn’t know about it until I looked for it”* (Termination)

*Domain 1: Experiences during counseling (*Table 1)

### Barriers to decision making related to prenatal counseling

One theme, areas for improvement in counseling, was dominant. Five subthemes were identified: *insufficient discussion about either termination or hospice birth, perceived lack of urgency, inconsiderate behaviors, lack of acknowledgment of impact of fetal CHD on family unit,* and *insufficiently specific or transparent language.* Regarding *insufficient discussion*, one participant noted: “I had to… ask for that [surgical option for termination] and really push for what does that actually mean.” Others perceived a *lack of urgency* regarding access to multidisciplinary counseling: “I wish they [multidisciplinary counseling] would be more easily accessible that you can talk right away to a doctor that would do a surgery.” Some participants noted *inconsiderate* *behaviors* from counseling providers: “[counseling provider] gave us a newborn onesie that said, like little heart warrior… I found that offensive and distasteful...”

Many participants noted areas for improvement in counseling content, such as the *lack of acknowledgement* of how other family members may be affected: “Before making that decision [termination], there was no real mention of how, from what I remember anyway, her [sibling’s] life could potentially be impacted.” Another content-related subtheme was the *desire for more specific or transparent language:* “After going through a surgical D&E termination, I do feel like my understanding of what [the specific process of termination] was going to happen was obviously different than actually what happened…”

### Facilitators to decision making related to prenatal counseling

The dominant facilitator was perceived strengths of counseling practices. Six subthemes were identified: *value of multidisciplinary counseling, transparency about outcomes, respect for patient autonomy, patience and understanding, nonverbal communication practices,* and *use of written materials.* Many participants noted the *value of multidisciplinary counseling*: “…there was 10 different people… And then if there was questions that somebody didn’t know, somebody else would jump in, and that was helpful.”

Participants also appreciated *transparency about outcomes*: “I needed the facts to be able to understand, I guess, how serious it [the diagnosis] was.” Participants appreciated language that *respected their autonomy*: “if somebody leaned a certain way, I think it maybe would have given us false hope.” Some participants noted the importance of *nonverbal communication practices*, for instance, the physical location and setting for counseling and particularly appreciated *written* *tools*: “…and [the cardiologist] drawing not just this is what the current state is, but what would happen if we had the surgery and that helped me a lot.”

### Context-dependent facilitators or barriers to decision making related to prenatal counseling

Two themes, patient attributes specifically affecting counseling and views on provider recommendation contained subthemes that could act as either barriers or facilitators, depending on individual context and preferences. Barrier-focused subthemes included *shock and information overload, previous knowledge or attitudes toward termination or hospice,* and *undesired bias*. Most participants noted that they were initially *shocked and overwhelmed* after receiving a diagnosis of fetal CHD, which was a barrier to processing information: “I don’t know if I’ve repressed or suppressed some of these memories...” Several participants noted that their *previous knowledge or attitudes toward termination* served as a barrier to their decision. A participant also described their *previous understanding of hospice* as a barrier to their decision: “I think my immediate reaction to it [hospice] was that it was so passive, and just almost non-committal.” Finally, some participants described *undesired bias* against their final decision to pursue hospice birth: “I could have ended up delivering with her [midwife who was not supportive of decision] and that would have been very hard.”

However, in other contexts, these subthemes emerged as facilitators, including *previous knowledge or attitudes toward hospice birth*, and *desire for provider recommendation*. For some participants who chose hospice birth, *previous knowledge about hospice* was a facilitator to hospice birth counseling: “I had a couple of family members that had been on hospice... I knew what hospice was and what it entailed and everything.” Finally, although most participants described a preference for, and experience of, neutral counseling, a few participants expressed a *desire for provider recommendation*: “sometimes I wish that there was more of a bias because I felt like it would make me feel like I didn’t have to make this decision.”

*Domain 2: Individual factors affecting decision making (***Table 2**)

Six themes emerged regarding individual considerations in decision making: emotions affecting decision making following counseling, value for reproductive autonomy (i.e., the ability to make informed decisions about reproduction free from coercion or constraint) [26], family factors, internal factors, outside input, and desire for resources. First, regarding emotions impacting decision making, the subthemes of *guilt* or *uncertainty* were common. One participant described *guilt* in the context of their living child: “of course I would do whatever for her [living daughter], so then it tore me to be like, well, I’m not going to do whatever for him [fetus with CHD]…” Another participant explained how *uncertainty* was driven by institutional counseling practices: “…after my initial consultation at [Institution 1]…”Oh, it was just a couple of surgeries and we can be all good.” I think after [consultation at Institution 2] is when I was like, okay…it’s not good…” Many participants who experienced termination described a *fear of stigma* for their decision, but those who chose hospice experienced stigma also: “our neighbor…was not very supportive. And I didn’t go into details with everybody...”

Regarding the theme of value for reproductive autonomy, analysis revealed subthemes of *reproductive justice* and *shifts in opinion about termination*. Specifically, reproductive justice refers to the emphasis on the human right to maintain bodily autonomy, have children or not have children [27]. With regards to reproductive justice, a participant who chose hospice still described her *appreciation for having the option of termination*: “even though I didn’t do it [termination]… it was still an option that I could have chosen...” Another participant described *opinion shifts* in a close family member due to their experience: “This experience of mine has really completely changed his [family member’s] perspective of that [termination]… he was very clear from the get-go like that I should make the choice that’s right for me.”

Regarding the theme of family factors that affect decision making, six subthemes were identified: *spousal disagreement, baby’s suffering, baby’s long-term outcomes, effects on living children, financial consequences,* and *insurance and access to care.* Regardless of whether a participant experienced termination or hospice, there were cases of *spousal disagreement*. In these cases, the birthing parent was the final decision maker, as one participant who elected for hospice birth described: “My husband was leaning more towards the termination route. But my body, so I told him like, I’m not doing that.” One participant also summarized their desire for information on *long-term outcomes*: “…about their IQ level, like, how much is it going to go down? Is he going to be able to do certain things?”

Several participants discussed the *effects of caring for a child with CHD on other living children*: “We were not sure how we were going to take care of one while the other one needed some help…. it’s hard to find help with the kids even if you can afford to pay.” Many participants described financial consequences of having a child with a chronic disease: “…then if you need help, how will insurance cover it [healthcare costs], we’ll be paying out of pocket. I think monetary, I mean, we’re all concerned about that I think.” Another participant described *insurance* as a major consideration in decision making: “I’m the one that has our insurance…so then if I wasn’t working, where would our insurance come from?”

Many reflected about internal factors which affected their decision making. These subthemes included *personal risk tolerance, religious beliefs,* and *shifts in personal priorities*. One participant described their own limited *risk tolerance* for poor outcomes: “…just knowing that there were those super negative options, that was enough…” Another participant who elected for hospice birth described how their *religious beliefs* shaped their decision: “…in Judaism, we say that the baby is not a life until it’s born, until part of its space is out of the body.” Multiple participants described *shifts in personal priorities* that shaped their decision: “…maybe it is selfish, but I didn’t want to quit my job and live in a hospital caring for a sick child.”

Most participants sought outside input in their decision making. The reported sources of input included *family, friends or community*, and *blogs and social media.* As an example of *community* support, one participant noted: “and his [priest’s] stance was well, just because there’s medical technology and advances doesn’t mean that you should use them on people.” Multiple families sought information about lived experiences among children with CHD, primarily through *social media*: “But I just knew that it [surgical intervention] wasn’t what I wanted for my child in the sense of some of the detail that people put on those blogs and journal type entries…”

Multiple participants noted a desire for resources pertaining to others’ *experiences with termination or hospice birth* and *experiences of children living with CHD.* One participant noted: “I think it would be good to have talked to another family that went through it [termination] and just to see their reasons because maybe they’re going to be different.” Overall, participants chose to pursue online content to aid with decision making, over meetings with families who chose postnatal surgery: “maybe have a site with resources… having this information right away… would make I think the decision much faster.”

## Discussion

This analysis exploring the lived experiences of birthing parents who underwent termination or chose a hospice birth for a fetus with severe CHD reveals four key findings. First, their value for reproductive autonomy and the provision of information about all options available. This desire for information was present regardless of personal beliefs about termination or hospice birth. Second is the importance of considering biases during counseling and leveraging neutral language, which was the preference of most birthing parents. Third is the need for discussion on the effects of fetal CHD on the family unit, finances, and personal priorities. Fourth is the importance of providing information about local resources that can add support for families that are faced with this decision. Although these findings may seem intuitive for practitioners with formal or practical training in reproductive choice counseling and/or palliative care, such skills are not a routine aspect of all subspecialty training.

Despite the majority of birth parents in this study citing a desire for provision of all information through a neutral lens, previous studies have quantified how cardiologists’ personal beliefs influence whether termination or hospice birth are mentioned during counseling for severe fetal CHD [28,29], and some parents perceive that surgery can be over-emphasized [30]. Inclusion of palliative care specialists during counseling ensures not only that all management options are presented, but also – given the dedicated training that palliative care specialists have in communication and counseling – that these options are presented as neutral choices. Yet, a recent benchmarking survey revealed that only about 40% of major cardiac centers in North America conduct joint counseling with cardiologists and palliative care specialists [31].

A key aspect of the palliative care approach is a focus on listening, learning about, and allowing for recommendations based on the patient’s values. For example, while most participants preferred neutral presentation of management options, some participants had a desire for the provider to make a recommendation. In such an instance, it may be appropriate to ask parents whether hearing a recommendation from the medical team is important to them. If so, the clinician’s recommendation should consider the severity and long-term outcomes of the CHD, as well as the parents’ values. However, the clinician should also make clear that parents may choose to reject such recommendations and still be fully supported by the clinician. Communication trainings such as VitalTalk (https://www.vitaltalk.org/) may help counseling providers to gain more confidence in this style of communication. Future research should also include provider perspectives on the challenges of avoiding bias while still supporting each family’s wishes. Such data would provide a comprehensive view for future training curricula in fetal cardiac counseling, which remains a major focus area in the field [6–8].

This analysis identified a key subtheme – value for counseling beyond clinical outcomes, especially the potential long-term consequences on the family unit. This subtheme was novel and relatively unique to this study, possibly because previous prenatal counseling studies were primarily conducted among parents who elected for surgical palliation [12–16]. However, Carlsson and Mattsson’s work [20,32], conducted with a Swedish population that included termination, found similar subthemes to this analysis, such as a desire for more information about impacts on family life. Ultimately, we find that the unique subtheme for value for counseling beyond clinical outcomes was better supported by studies among families that had already experienced postnatal surgical palliation, including the time away from work to care for a child with CHD [33], costs of care including forgone income and the loss of insurance if one parent needed to exit the workforce [34], and effects on the parents’ mental health [35] and other children [36]. Counseling providers should inform parents of the available supports that can offset these challenges, such as availability of child life support for siblings, sibling childcare, and subsidized lodging near the surgical hospital, while also considering that the healthcare and social welfare systems in the United States are poorly designed to support patients with chronic disease compared to other high-income countries [37].

Previous studies conducted in the United States pertaining to decision making among individuals who chose termination due to fetal CHD have primarily used a quantitative approach. Although studies outside the United States have found parent ethnicity and religion to be strongly associated with termination [38,39], a more recent study found no significant association between religion and termination after adjustment for CHD severity [40]. Our analysis similarly found that individuals of different religion, religiosity, and social experiences chose termination, although our study design did not allow for estimation of associations. Similarly, Delaney *et al* undertook a qualitative approach [30], conducting focus groups among pregnant individuals who chose termination, palliative care, or surgery. Their analysis revealed some themes like ours, such as reproductive autonomy (described by Delaney *et al* as “Incongruent discussion of treatment options”) and the importance of consequences on the family unit. Our work extends prior work by identifying novel themes pertaining to barriers and facilitators in counseling, which have practical applications for clinical practice.

A primary strength of this analysis was the existing relationship between the interviewer and the participants, which allowed us to recruit and conduct sensitive, in-depth interviews. However, several limitations warrant consideration. First, most of the participants identified as non-Hispanic White race, married, employed, and with at least a college-level education. The experiences faced by patients of lower socioeconomic status, or unmarried patients, are not well-represented. These less-resourced populations may have experienced access barriers to multidisciplinary care and/or termination or hospice birth services, highlighting a key knowledge gap for future studies. Second, many participants received counseling during the COVID-19 pandemic, which itself introduced stressors that may have contributed to recall bias. In addition, the COVID-19 pandemic may have delayed or limited multidisciplinary counseling. This barrier could have exaggerated the subtheme of a perceived lack of urgency. An additional source of recall bias includes the time gap between receipt of multidisciplinary counseling and enrollment in the study, a tradeoff we accepted to appropriately account for bereavement time. Third, many decisions for pregnancy termination due to fetal CHD occur before patients receive multidisciplinary counseling or even counseling from a single clinician; these patients were not represented in our purposive sample of patients who were referred to this specialized clinical context.

Fourth, we acknowledge that this study took place at a single center in Illinois, where reproductive autonomy is protected by state law, and many patients have ready access to comprehensive care such as the multidisciplinary counseling outlined in this study. Although this context is a strength to the analysis, as all options were available to participants, future work must explore other contexts, such as: situations in which termination cannot be discussed or provided without substantial burden to patients due to legal restrictions, situations where patients do not have access to multidisciplinary counseling, or international settings where attitudes toward termination and hospice birth may differ [41,42].

## Conclusion

Families who choose termination or hospice due to fetal CHD are an understudied group who navigate uniquely complex experiences, which may include guilt, fear of stigma, spousal disagreement, and suboptimal counseling. Yet these individuals expressed clear desires for receiving comprehensive information beyond clinical outcomes, including wider implications for their finances, personal priorities, and family. The central importance of reproductive autonomy underscores the need for training in equitable, patient-centered counseling techniques that address all options with empathy and neutrality.

## Supporting information

S1 Document Interview Guide. (DOCX)

S2 Document Standards for Reporting Qualitative Research (SRQR) checklist. (DOCX)

## References

[1] Hoffman JIE, Kaplan S. The incidence of congenital heart disease. J Am Coll Cardiol. 2002;39(12):1890–900. doi: 10.1016/s0735-1097(02)01886-7 12084585

[2] Oster ME, Lee KA, Honein MA, Riehle-Colarusso T, Shin M, Correa A. Temporal trends in survival among infants with critical congenital heart defects. Pediatrics. 2013;131(5):e1502-8. doi: 10.1542/peds.2012-3435 23610203 PMC4471949

[3] Valdes C, Chiu JS, Ronai C, Haxel CS. Association Between Institutional Services and Counseling Options in Fetal Cardiology. Prenat Diagn. 2026;46(3):382–9. doi: 10.1002/pd.70094 41697259

[4] Bonnet D. Impacts of prenatal diagnosis of congenital heart diseases on outcomes. Transl Pediatr. 2021;10(8):2241–9. doi: 10.21037/tp-20-267 34584895 PMC8429871

[5] Allan LD, Huggon IC. Counselling following a diagnosis of congenital heart disease. Prenat Diagn. 2004;24(13):1136–42. doi: 10.1002/pd.1071 15614846

[6] Pinto NM, Morris SA, Moon-Grady AJ, Donofrio MT. Prenatal cardiac care: Goals, priorities & gaps in knowledge in fetal cardiovascular disease: Perspectives of the Fetal Heart Society. Prog Pediatr Cardiol. 2020;59:101312. doi: 10.1016/j.ppedcard.2020.101312 33100800 PMC7568498

[7] Donofrio MT, Moon-Grady AJ, Hornberger LK, Copel JA, Sklansky MS, Abuhamad A, et al. Diagnosis and treatment of fetal cardiac disease: a scientific statement from the American Heart Association. Circulation. 2014;129(21):2183–242. doi: 10.1161/01.cir.0000437597.44550.5d 24763516

[8] Keelan JA, Moon Grady AJ, Arya B, Donofrio MT, Schidlow DN, Tacy TA, et al. Current State of Fetal Heart Disease Counseling and Training: Room for Improvement?: Endorsed by the Fetal Heart Society. Pediatr Cardiol. 2022;43(7):1548–58. doi: 10.1007/s00246-022-02882-4 35380215

[9] Almeida SLDM, Tuda LTS, Dias MB, Carvalho LIAD, Estevam TLL, Novelleto ALMT. Family counseling after the diagnosis of congenital heart disease in the fetus: scoping review. Healthcare. 2023. 10.3390/healthcare11212826 PMC10647570 37957971

[10] Dandy S, Wittkowski A, Murray CD. Parents’ experiences of receiving their child’s diagnosis of congenital heart disease: A systematic review and meta-synthesis of the qualitative literature. Br J Health Psychol. 2024;29(2):351–78. doi: 10.1111/bjhp.12703 37968248

[11] Kovacevic A, Wacker-Gussmann A, Bär S, Elsässer M, Mohammadi Motlagh A, Ostermayer E. Parents’ perspectives on counseling for fetal heart disease: what matters most?. J Clin Med. 2022;11(1). 10.3390/jcm11010278 PMC8745975 35012018

[12] Houlihan TH, Combs J, Smith E, Coulter E, Figueroa L, Falkensammer C, et al. Parental Impressions and Perspectives of Efficacy in Prenatal Counseling for Single Ventricle Congenital Heart Disease. Pediatr Cardiol. 2024;45(3):605–13. doi: 10.1007/s00246-023-03355-y 38112807 PMC10891191

[13] Marshall E, Gramszlo C, Perez Ramirez A, Kazak AE, Shillingford AJ, Ortinau CM, et al. Interpersonal relationships after prenatal diagnosis of congenital heart disease: Social stressors and supports. J Perinatol. 2025;45(7):907–13. doi: 10.1038/s41372-025-02250-z 40050404 PMC12317836

[14] Harris KW, Brelsford KM, Kavanaugh-McHugh A, Clayton EW. Uncertainty of Prenatally Diagnosed Congenital Heart Disease: A Qualitative Study. JAMA Netw Open. 2020;3(5):e204082. doi: 10.1001/jamanetworkopen.2020.4082 32369178 PMC7201310

[15] Woo JL, Gandhi R, Burton S, Sivakumar A, Spiewak S, Wakulski R, et al. Perspectives of Challenges in Counseling for Congenital Heart Defects. Pediatr Cardiol. 2025;46(4):947–55. doi: 10.1007/s00246-024-03520-x 38907869 PMC11903537

[16] Rempel GR, Cender LM, Lynam MJ, Sandor GG, Farquharson D. Parents’ perspectives on decision making after antenatal diagnosis of congenital heart disease. J Obstet Gynecol Neonatal Nurs. 2004;33(1):64–70. doi: 10.1177/0884217503261092 14971554

[17] Bertaud S, Lloyd DFA, Sharland G, Razavi R, Bluebond-Langner M. The impact of prenatal counselling on mothers of surviving children with hypoplastic left heart syndrome: A qualitative interview study. Health Expect. 2020;23(5):1224–30. doi: 10.1111/hex.13103 32671929 PMC7696135

[18] Bratt E-L, Järvholm S, Ekman-Joelsson B-M, Mattson L-Å, Mellander M. Parent’s experiences of counselling and their need for support following a prenatal diagnosis of congenital heart disease--a qualitative study in a Swedish context. BMC Pregnancy Childbirth. 2015;15:171. doi: 10.1186/s12884-015-0610-4 26276642 PMC4537590

[19] Schneider K, Bousi D, Stressig R. Impact of interdisciplinary counselling for parental decision-making in cases of pregnancies with prenatally diagnosed CHD. Cardiology in the Young. 2023;33(7):1172–6. 35915979 10.1017/S104795112200213X

[20] Carlsson T, Bergman G, Wadensten B, Mattsson E. Experiences of informational needs and received information following a prenatal diagnosis of congenital heart defect. Prenat Diagn. 2016;36(6):515–22. doi: 10.1002/pd.4815 26991536 PMC5074242

[21] Janssen HJ, Cuisinier MC, de Graauw KP, Hoogduin KA. A prospective study of risk factors predicting grief intensity following pregnancy loss. Arch Gen Psychiatry. 1997;54(1):56–61. doi: 10.1001/archpsyc.1997.01830130062013 9006401

[22] Lasker JN, Toedter LJ. Acute versus chronic grief: the case of pregnancy loss. Am J Orthopsychiatry. 1991;61(4):510–22. doi: 10.1037/h0079288 1746627

[23] https://www.supremecourt.gov/opinions/21pdf/19-1392_6j37.pdf. Dobbs v. Jackson 2021 [.

[24] Sabbath EL, McKetchnie SM, Arora KS, Buchbinder M. US Obstetrician-Gynecologists’ Perceived Impacts of Post-Dobbs v Jackson State Abortion Bans. JAMA Netw Open. 2024;7(1):e2352109. doi: 10.1001/jamanetworkopen.2023.52109 38231510 PMC10794934

[25] Smith MH, Young D, Dismer H, Diskin V, Hasselbacher L, Rivlin K, et al. Clinic Adaptations and Changes in Abortion Use After Dobbs in Illinois, July 2021-June 2023. Am J Public Health. 2026;116(3):359–67. doi: 10.2105/AJPH.2025.308273 41100803 PMC12895905

[26] Upadhyay UD, Dworkin SL, Weitz TA, Foster DG. Development and validation of a reproductive autonomy scale. Stud Fam Plann. 2014;45(1):19–41. doi: 10.1111/j.1728-4465.2014.00374.x 24615573

[27] Ross L, Solinger R. Reproductive justice: An introduction. Univ of California Press. 2017.

[28] Haxel CS, Ronai C, Martens AM, Lim CC, Pinto N, Chiu JS. Evaluating how beliefs among pediatric cardiology providers may affect fetal cardiac counseling—A national perspective. Prenatal Diagnosis. 2025;45(6):752–63. 39613724 10.1002/pd.6706

[29] Woo JL, Swanson TM, Maskatia SA, Morris SA, Lantos JD, Patel A. Associations of Physician Perspectives, Personal Choices, and Counseling for Severe Congenital Heart Defects. Prenat Diagn. 2025;45(11):1402–8. doi: 10.1002/pd.6901 40988087 PMC12530347

[30] Delaney RK, Pinto NM, Ozanne EM, Brown H, Stark LA, Watt MH, et al. Parents’ decision-making for their foetus or neonate with a severe congenital heart defect. Cardiol Young. 2022;32(6):896–903. doi: 10.1017/S1047951121003218 34407894 PMC8857292

[31] Patel S, McBrien A, Michelfelder E, Kavanaugh-McHugh A, Samples S, Laternser C, et al. Current trends in fetal cardiology: results from an international benchmarking survey of fetal cardiac programs through the fetal heart society research collaborative. Pediatric Cardiol. 2025;:1–11. 10.1007/s00246-025-03895-5 PMC12901083 40439930

[32] Carlsson T, Mattsson E. Emotional and cognitive experiences during the time of diagnosis and decision-making following a prenatal diagnosis: a qualitative study of males presented with congenital heart defect in the fetus carried by their pregnant partner. BMC Pregnancy Childbirth. 2018;18(1):26. doi: 10.1186/s12884-017-1607-y 29329527 PMC5767070

[33] Connor JA, Kline NE, Mott S, Harris SK, Jenkins KJ. The meaning of cost for families of children with congenital heart disease. J Pediatr Health Care. 2010;24(5):318–25. doi: 10.1016/j.pedhc.2009.09.002 20804952

[34] Pickles DM, Keller K. The Cost of Complex Congenital Heart Disease in the US. Additional Ventures. 2024.

[35] McClung N, Glidewell J, Farr SL. Financial burdens and mental health needs in families of children with congenital heart disease. Congenit Heart Dis. 2018;13(4):554–62. doi: 10.1111/chd.12605 29624879 PMC6105538

[36] Tay J, Widger K, Steele R, Stremler R, Pole JD. Examining emotional and behavioural trajectories in siblings of children with life-limiting conditions. BMC Palliat Care. 2024;23(1):205. doi: 10.1186/s12904-024-01535-y 39129022 PMC11318302

[37] Schoen C, Osborn R, How SK, Doty MM, Peugh J. In chronic condition: experiences of patients with complex health care needs, in eight countries. Health Affairs. 2008;27(Suppl1):w1–16. 10.1377/hlthaff.28.1.w1 19008253

[38] Gendler Y, Birk E, Tabak N, Koton S. Factors that influence parents’ decision-making regarding termination of pregnancy after prenatal diagnosis of fetal congenital heart disease. J Obstet Gynecol Neonatal Nurs. 2021;50(4):475–84. doi: 10.1016/j.jogn.2021.04.002 33991490

[39] Chenni N, Lacroze V, Pouet C, Fraisse A, Kreitmann B, Gamerre M, et al. Fetal heart disease and interruption of pregnancy: factors influencing the parental decision-making process. Prenat Diagn. 2012;32(2):168–72. doi: 10.1002/pd.2923 22418961

[40] Perez MT, Bucholz E, Asimacopoulos E, Ferraro AM, Salem SM, Schauer J, et al. Impact of maternal social vulnerability and timing of prenatal care on outcome of prenatally detected congenital heart disease. Ultrasound Obstet Gynecol. 2022;60(3):346–58. doi: 10.1002/uog.24863 35061294

[41] Kamranpour B, Noroozi M, Bahrami M. A qualitative study exploring the needs related to the health system in women with experience of pregnancy termination due to fetal anomalies in Iran. BMC Pregnancy Childbirth. 2020;20(1):573. doi: 10.1186/s12884-020-03274-3 32993553 PMC7526095

[42] Tewani K, Singh R, Wendy CPY, Jia Huan H, Jayagobi P, Teo I. Understanding the experiences of mothers receiving perinatal palliative care: a qualitative study. Palliat Med. 2023;37(9):1379–88. doi: 10.1177/02692163231171182 37132995

