# Supplementary material for: Parental perspectives of prenatal counseling and decision making for termination or hospice birth due to fetal congenital heart defects
Source: PLoS One. 2026 Jul 30;21(7):e0353868. doi: 10.1371/journal.pone.0353868 (PMC13423054; doi:10.1371/journal.pone.0353868)
Supplement: S1 Document — (DOCX) [file pone.0353868.s001.docx]

**Supplemental Document 1: Interview Guide**

| Project Title | Decision-making for termination or hospice birth plan due to severe fetal congenital heart disease: A qualitative study |
| --- | --- |
| IRB Protocol Number | IRB 2023-6213 |

Interviewer instructions are listed in blue.

- The interviewer will invite the interviewee to ask any remaining questions about the **information sheet.**
- The interviewer will invite participants to turn off their cameras, if they so desire, before recording commences.

We are recording now. Thank you for agreeing to meet with me today. I would like to start with some questions about your prenatal counseling experience.

**Topic 1: Prenatal Counseling Experience**

**Subtopic 1A: Immediate post-diagnosis experience**

1. Could you describe your most prominent emotion after receiving a diagnosis of CHD in your fetus?
   1. Were there any factors that impacted your initial feelings and thoughts upon first receiving the diagnosis of CHD in your baby? *Prompts:*
      1. *Shock after the diagnosis*
      2. *Baseline stress levels*
      3. *Too much medical terminology*
      4. *People talking too fast*
   2. Did that primary emotion change after learning more about CHD from the cardiologist? After meeting with the palliative care clinician?

**Suptopic 1B: Counseling Experience**

1. How would you describe the quality of the prenatal counseling you received for the diagnosis of CHD in your fetus overall?
2. I would like to hear about how the care team helped you understand the diagnosis and your management options:
   1. Did you find that counseling from all the specialists you spoke with was necessary to fully understand the diagnosis and its management?
   2. Did you feel confident in the accuracy of the information that the medical team provided to you?
   3. Was there a person or specialist whose input contributed most to your family’s understanding or final decision? What did they say or do that was helpful? *Prompt:*
      1. *For instance, was there a particular clinician who helped you understand your child’s lesion?*
   4. Were there additional resources that were provided to you and your family that you found to be particularly helpful (or unhelpful)?
      1. *For example, Facebook groups, peer support, etc.*
3. I would like to hear about how your interactions with the care team around the baby’s diagnosis made you feel:
   1. Was there anything said or done during counseling that made you feel uncomfortable, or made you feel less supported after the diagnosis was made?
   2. Did you feel that all the specialists took your family and life situation into account when talking with you and your partner? *Prompt:*
      1. *For instance, was there any experience with illness in a child or family member?*

**Subtopic 1C: Presentation of Management Options**

I would like to talk a little more about how pathways for care were presented to you.

1. Do you recall if any clinician talked with you about different clinical pathways for the care of your child? If so, who, and what pathways did they share were options for your child?
   1. Was interruption of pregnancy presented to you as an option to manage the baby’s’ CHD? *Prompts: by whom, when*
      1. Was interruption ever a realistic option for your family? Why or why not?
      2. If you were the provider, how would you have brought up this option: the same or differently?
      3. If you indicated that this was not an option you would consider, did the specialists respect that decision going forward?
   2. Was a hospice birth plan or non-surgical pathway discussed during the pregnancy as an option to manage the baby’s CHD? *Prompts: by whom, when*
      1. What did you know or understand about hospice before this was offered as a pathway for your baby?
      2. What were your immediate feelings about this option when it was first brought up? What did you think that it meant?
      3. Did these feelings change as you learned more about what a hospice birth plan entails?
      4. Was a hospice birth plan ever a realistic option for your family? Why or why not?
      5. If you were the provider, how would you have brought up this option: the same, or differently?
      6. If you ever sought a second opinion somewhere else prenatally, did the other institution offer a hospice birth plan as an option for the baby after birth?
2. Did you sense any bias from your providers in which pathway was "best"? Was this helpful or not in coming to your decision? Would hearing how they thought best to proceed be welcome or unwelcome?

**Topic 2: Decision-making about management**

**Subtopic 2A: Decision-making for all participants**

1. How long after you received the CHD diagnosis did it take for you and your family to come to a decision about how to manage the rest of the pregnancy?
2. Do you feel that your providers provided you enough information to make the decision? Did you feel able to ask additional questions and if so, were they answered? Looking back, is there anything you wish they had told you?
3. What were the primary factors that influenced your decision? *Prompts:*
   1. *Pain or suffering that your baby might experience*
   2. *The risks and benefits of the surgical pathway for yourself*
   3. *A co-existing abnormality*
   4. *The possibility for future neurologic or developmental abnormalities*
   5. *The potential need for heart transplant at some point in your child’s life*
   6. *Length of hospitalizations or disruption caused by these hospitalizations to your family’s life*
   7. *The impact on other children or family members*
   8. *Faith*
   9. *Gut feeling*
4. What considerations for yourself and your pregnancy impacted your decision? *Prompts:*
   1. *Finances or insurance*
   2. *Spouses’ career*
   3. *Other children*
5. Sometimes families will find that the ‘unknowns’ and ‘unpredictability’ can make decision making challenging.  How did you grapple with the uncertainty and how did it impact your decision making, if at all?
   1. Did you seek advice from anyone (family or non-family members, online communities like Facebook groups, etc.) before making your decision?
6. Did you feel supported or judged by providers, or others, **after** you shared the decision that you made for your family? If so, how?
7. What do you think are the best ways to support families that made decisions like yours?

**Subtopic 2B: Decision-making (only for families who elected for a hospice birth plan)**

1. What were some of the considerations you had to make in choosing a hospice birth plan? *Prompts:*
   1. *Location of delivery*
      1. *Was the option of delivering at a hospital closer to home, with accommodation of a hospice birth plan, presented to you?*
   2. *Financial considerations*
      1. *Insurance changes*
      2. *Job changes*
   3. *Changes in family life*
2. Did you share your decision for a hospice birth plan with your family and close friends? If yes, did you have to explain what a hospice birth plan is, and how/why you chose this plan?
3. What ultimately made you and your spouse choose a hospice birth plan over surgical palliation?

**Subtopic 2C: Peri-/postnatal experience (only for families who elected for a hospice birth plan)**

1. Could you describe your birth experience?
   1. Did you feel that your birth plan was followed, and your wishes honored?
2. Can you tell me about the time you had with your baby?
   1. Can you share with me if you knew that the end of your baby’s life was getting closer and what that was like?
   2. Did you feel at any time your baby experienced discomfort? If so, how was that discomfort addressed? Was the intervention effective?
   3. Was the time you were able to spend with your baby during their life at the hospital or at home? Was that timeframe what you had expected based on guidance you were given while creating your birth plan? If not, was it longer or shorter than expected, and how did that make you feel?
   4. Did you feel as prepared as possible for your baby’s death? If not, what do you wish you could have known?
3. What surprised you during this experience? *Prompt:*
   1. *Were there challenges that you or your family experienced, that you didn’t expect?*
4. Do you remember interacting with anyone from the medical team? If so, were those interactions supportive? If not, how could they have been improved?
5. Knowing what you know now, would you have changed any part of your hospice birth plan or experience?

1. How could the prenatal team have better prepared you and your family for the hospice birth experience?

**Topic 3: Closing remarks**

1. What additional things would you like for providers or families to know to help improve this sort of challenging decision making for future families?  Is there anything else you would like to share around anything we discussed today before we finish?

- Stop recording
- Thank participant for time
- Provide contact information if questions/comments arise after interview (see **information sheet)**
- Provide support resources (see **information sheet**)
- Where applicable, interviewer will immediately move Zoom recording to the shared drive and delete from the cloud.

**Supplemental Document 2: Standards for Reporting Qualitative Research (SRQR)* checklist**

|  |  |  |
| --- | --- | --- |
|  | <http://www.equator-network.org/reporting-guidelines/srqr/> |  |
|  |  | **Page/line no(s).** |
| **Title and abstract** | |  |
|  | **Title** - Concise description of the nature and topic of the study Identifying the study as qualitative or indicating the approach (e.g., ethnography, grounded theory) or data collection methods (e.g., interview, focus group) is recommended | Title page |
|  | **Abstract** - Summary of key elements of the study using the abstract format of the intended publication; typically includes background, purpose, methods, results, and conclusions | Lines 29-50 |
|  |  |  |
| **Introduction** | |  |
|  | **Problem formulation** - Description and significance of the problem/phenomenon studied; review of relevant theory and empirical work; problem statement | Lines 64-67 |
|  | **Purpose or research questio**n - Purpose of the study and specific objectives or questions | Lines 68-70 |
|  |  |  |
| **Methods** | |  |
|  | **Qualitative approach and research paradigm** - Qualitative approach (e.g., ethnography, grounded theory, case study, phenomenology, narrative research) and guiding theory if appropriate; identifying the research paradigm (e.g., postpositivist, constructivist/ interpretivist) is also recommended; rationale** | Lines 96-114 |
|  | **Researcher characteristics and reflexivity** - Researchers’ characteristics that may influence the research, including personal attributes, qualifications/experience, relationship with participants, assumptions, and/or presuppositions; potential or actual interaction between researchers’ characteristics and the research questions, approach, methods, results, and/or transferability | Lines 99-102, Lines 111-113 |
|  | **Context** - Setting/site and salient contextual factors; rationale** | Lines 74-91 |
|  | **Sampling strategy** - How and why research participants, documents, or events were selected; criteria for deciding when no further sampling was necessary (e.g., sampling saturation); rationale** | Lines 77-83 |
|  | **Ethical issues pertaining to human subjects** - Documentation of approval by an appropriate ethics review board and participant consent, or explanation for lack thereof; other confidentiality and data security issues | Lines 126-129 |
|  | **Data collection methods** - Types of data collected; details of data collection procedures including (as appropriate) start and stop dates of data collection and analysis, iterative process, triangulation of sources/methods, and modification of procedures in response to evolving study findings; rationale** | Lines 103-114 |
|  | **Data collection instruments and technologies** - Description of instruments (e.g., interview guides, questionnaires) and devices (e.g., audio recorders) used for data collection; if/how the instrument(s) changed over the course of the study | Lines 72-77 |
|  | **Units of study** - Number and relevant characteristics of participants, documents, or events included in the study; level of participation (could be reported in results) | Lines 125-126 |
|  | **Data processing** - Methods for processing data prior to and during analysis, including transcription, data entry, data management and security, verification of data integrity, data coding, and anonymization/de-identification of excerpts | Lines 116-124 |
|  | **Data analysis** - Process by which inferences, themes, etc., were identified and developed, including the researchers involved in data analysis; usually references a specific paradigm or approach; rationale** | Lines 116-124 |
|  | **Techniques to enhance trustworthiness** - Techniques to enhance trustworthiness and credibility of data analysis (e.g., member checking, audit trail, triangulation); rationale** | Lines 116-124 |
|  |  |  |
| **Results/findings** | |  |
|  | **Synthesis and interpretation** - Main findings (e.g., interpretations, inferences, and themes); might include development of a theory or model, or integration with prior research or theory | Lines 130-281 |
|  | **Links to empirical data** - Evidence (e.g., quotes, field notes, text excerpts, photographs) to substantiate analytic findings | Lines 130-281  Tables 1 and 2 |
|  |  |  |
| **Discussion** | |  |
|  | **Integration with prior work, implications, transferability, and contribution(s) to the field -** Short summary of main findings; explanation of how findings and conclusions connect to, support, elaborate on, or challenge conclusions of earlier scholarship; discussion of scope of application/generalizability; identification of unique contribution(s) to scholarship in a discipline or field | Lines 294-302  Lines 322-334 |
|  | **Limitations** - Trustworthiness and limitations of findings | Lines 337-344 |
|  |  |  |
| **Other** | |  |
|  | **Conflicts of interest** - Potential sources of influence or perceived influence on study conduct and conclusions; how these were managed | Title page |
|  | **Funding** - Sources of funding and other support; role of funders in data collection, interpretation, and reporting | Financial disclosure statement |
|  |  |  |
|  | *The authors created the SRQR by searching the literature to identify guidelines, reporting standards, and critical appraisal criteria for qualitative research; reviewing the reference lists of retrieved sources; and contacting experts to gain feedback. The SRQR aims to improve the transparency of all aspects of qualitative research by providing clear standards for reporting qualitative research. |  |
|  |  |  |
|  | **The rationale should briefly discuss the justification for choosing that theory, approach, method, or technique rather than other options available, the assumptions and limitations implicit in those choices, and how those choices influence study conclusions and transferability. As appropriate, the rationale for several items might be discussed together. |  |
|  |  |  |
|  | **Reference:** |  |
|  | O'Brien BC, Harris IB, Beckman TJ, Reed DA, Cook DA. **Standards for reporting qualitative research: a synthesis of recommendations.** *Academic Medicine*, Vol. 89, No. 9 / Sept 2014  DOI: 10.1097/ACM.0000000000000388 |  |
|  |  |  |
|  |  |  |
